# Impact of multidisciplinary care of diabetic foot infections for inpatients at Campbelltown Hospital

**DOI:** 10.1186/s12913-023-10119-0

**Published:** 2023-10-19

**Authors:** Timothy Choi, Uchechukwu Levi Osuagwu, Chau Tran, Krupali Bulsari, David Simmons

**Affiliations:** 1 School of Medicine, Western Sydney University, Campbelltown, NSW 2560 Australia; 2 Bathurst Rural Clinical School (BRCS), School of Medicine, Western Sydney University, Bathurst, NSW 2795 Australia; 3 Macarthur Diabetes Endocrinology Metabolism Services, Camden and Campbelltown Hospitals, Campbelltown, NSW 2560 Australia

**Keywords:** Diabetic foot Infections, Multidisciplinary team, High risk foot service, Multidisciplinary team, Length of stay, Surgical outcomes, South Western Sydney

## Abstract

**Background:**

Diabetic foot infection (DFI), including diabetic foot ulcer, is a serious complication of diabetes, particularly in the South Western Sydney (SWS) region where it is a leading cause of diabetes-related hospitalisations. Multidisciplinary team (MDT) involvement is effective at improving the health outcomes of DFI patients. This study investigated the impact of MDT (High Risk Foot Service, HRFS) on the length of stay and surgical outcomes of inpatients with DFI in a Sydney tertiary hospital.

**Method:**

A retrospective audit of electronic medical records of inpatient admissions for DFI at Campbelltown Hospital between January 2019 - December 2021, was performed. The main outcome of the study was MDT involvement, defined as having two or more specialities involved in the patient’s treatment. The other measured variables included length of stay (defined as the total duration from admission to discharge), and surgical outcomes including debridement, minor amputation, and major amputation.

**Results:**

Over the three years, 78 participants presented to the hospital for 89 unique DFI admissions. There were 24 admissions in 2019, 28 admissions in 2020, and 37 admissions in 2021, with MDT attendance showing a steady increase at 62.5%, 75.0% and 83.8% respectively. Patients with serious comorbidities such as chronic kidney disease were more likely to have MDT involvement (84.8% vs. 15.2%, P = 0.048). Imaging was more likely to be performed with MDT involvement (78.8% vs. 21.3%, p < 0.05). Comparing patients who received and did not receive MDT care, the mean HbA1c (%) (8.4 ± 2.0 vs. 8.2 ± 2.7, *P* = 0.701), median length of stay (LOS: 7.8, IQR 15.0 days vs. 4.8 IQR 7.9 days, *P* = 0.243) and rate of surgical outcomes (74.6% vs. 72.7%, *P* = 0.262) were similar. Patients who required major amputation had significantly longer LOS (24 days, IQR 21.5 vs. 5.2 days, IQR 13.0, *P* = 0.004) but similar HbA1c (*P* = 0.552) compared to those who had conservative intervention.

**Conclusion:**

Adopting an MDT approach was associated with more thorough investigation of DFI, with similar rates of surgical outcomes. Further research on the impacts of MDT on length of stay and surgical outcomes of DFI patients in other SWS hospitals is needed.

## Introduction/Background

Diabetic Foot Infection (DFI) is a major complication of diabetes, defined as the infection of any tissue below the malleoli in an individual with a history of diabetes [1]. It has become a significant health issue in Australia, particularly in South Western Sydney (SWS), where it is a leading cause of hospitalisation and major contributor to morbidity and mortality [2]. In Australia, DFI accounts for one in five of all diabetes-related hospital admissions [3]. These infections can have serious consequences for affected individuals, such as amputation, decreased mobility and independence, and a decreased quality of life [4]. They also place a significant burden on the healthcare system, with high associated costs and a need for ongoing medical care [4]. In SWS, diabetes-related hospitalisation rates are far higher than in other areas of New South Wales [2], and health outcomes tend to be poorer than the rest of the state [5].Clinical guidelines recommend high-risk patients, defined as those with two or more risk factors such as neuropathy, coronary artery disease, foot deformity, and/or a history of foot ulceration or amputation, to be referred to a multidisciplinary team (MDT) [6]. MDTs have been shown to be effective in improving the health outcomes of DFI patients. MDTs bring together a range of healthcare professionals such as, wound care nurses, vascular surgery, podiatry, endocrinology and infectious disease to provide comprehensive care to patients [6]. Several studies have demonstrated that MDT care is associated with lower rates of amputation, hospitalisation, and mortality, as well as improved quality of life and patient satisfaction [7–10].

The High Risk Foot Service (HRFS) MDT at Campbelltown Hospital, a major tertiary hospital in SWS, was significantly augmented in 2020 to include input from onsite consultants in vascular surgery, infectious disease, and wound care, with the purpose of optimising treatment and patient care. The aim of this retrospective audit was to investigate the impact of the recent expansion of the HRFS MDT at Campbelltown Hospital on inpatient length of stay (LOS) and surgical outcomes of high risk DFI patients. The results of this project may be used to assess the effectiveness of the HRFS MDT and identify areas for improvement.

## Methods

### Study setting and population

A retrospective audit was conducted of all inpatients at Campbelltown Hospital from January 1, 2019, to December 31, 2021, whose primary reason for admission was infected diabetic foot ulcer.

### Eligibility criteria

To identify eligible participants, patient data were initially filtered from the South Western Sydney Local Health District (SWSLHD) electronic medical records (eMR) using ICD-10 coding provided by the clinical information department to screen for patients who were admitted with the primary diagnosis of a foot infection. Inclusion criteria included age over 18 years, pre-existing diabetes, primary diagnosis of foot infection or ulcer, and patients who were admitted as an inpatient from the emergency department (ED) or directly from an outpatient clinic. A total of 91 potential participants were identified, of which 78 were included in the study after excluding 13 participants who did not meet the inclusion criteria or had incomplete medical records. A total of 89 unique admissions were used in the study.

### Data collection

An encrypted excel spreadsheet was used to collect data. Data on 52 variables were collected for each unique admission, including: (1) patient characteristics - age, sex, post-code, indigenous status, and comorbidities; (2) vitals data – body mass index (BMI), blood pressure (BP), heart rate (HR), respiratory rate (RR), and temperature on arrival to the emergency department; (3) clinical features of the foot ulcers – number of ulcers, location, probe to bone, offloading status, and Perfusion, Extent, Depth, Infection, Sensation (PEDIS) stage; (4) investigations performed - imaging, blood culture, wound culture, white cell count (WCC), erythrocyte sediment rate (ESR), and C-reactive Protein (CRP), which are investigations for DFI recommended by evidence-based guidelines [6]; (5) patient risk factors - established cardiovascular disease (CVD), foot deformity, peripheral neuropathy, duration of diabetes, glycaemic control, chronic kidney disease (including end stage renal disease), obesity, smoking status, alcohol intake, and diabetes medication. The PEDIS classification was developed by the International Working Group of the Diabetic Foot (IWGDF) as a universally accepted classification system developed primarily for DFI research [11]. Categorisation of the different variables is shown in Table 1.

**Table 1:** Patient characteristics (all cohort) and proportion with multidisciplinary team (MDT) involvement. Values are expressed as n (%) except where indicated

Variables	Frequency, n (%) (total n = 89)	MDT Involvement, n (%)
**Demographics**		
Age in years, mean (± SD)	62.3 (13.8)	
Sex		
Male	64 (71.9)	49 (55.1)
Female	25 (28.1)	18 (20.2)
Indigenous (yes)	3 (3.4)	2 (2.2)
Previously known to HRFS	62 (69.7)	47 (52.8)
**Comorbidities**		
Obesity (BMI > 30 kg/m2)	61 (68.5)	45 (50.5)
Hypertension (SBP > 130mmHg)	56 (62.9)	42 (47.2)
Established Cardiovascular Disease	63 (70.8)	49 (55.1)
Chronic Kidney Disease	46 (51.7)	39 (43.8)
End stage renal disease	10 (11.2%)	9 (10.1)
Existing Foot Deformity	37 (41.6)	28 (31.4)
Peripheral Neuropathy	59 (66.3)	44 (49.4)
HbA1c in %, mean (± SD)	8.4 (2.1)	8.2 (2.7)
HbA1c > 7% (53mmol/mol)	57 (67.9)	44 (49.4)
Current Smoker	18 (20.2)	15 (16.9)
Current Drinker	15 (16.9)	12 (13.4)
On Immunosuppression Therapy	1 (1.1)	1 (1.1)
**Laboratory results**		
Abnormal WCC (> 10 × 10^9/L)	53 (60.2)	43 (48.3)
Abnormal CRP (> 4.9 mg/L)	83 (93.3)	64 (71.9)
Blood Culture Performed	44 (49.4)	38 (42.7)
Wound Culture Performed	62 (69.7)	48 (53.9)
Imaging Performed	80 (89.9)	63 (70.8)
**Ulcer Characteristics**		
Forefoot Ulcer	63 (70.8)	
Hindfoot Ulcer	13 (14.6)	
Combination Ulcer	13 (14.6)	
Probing to Bone	21 (23.6)	
Offloading in Place	46 (51.7)	
**PEDIS Classification**		
Stage 1	2 (2.2)	2 (2.2)
Stage 2	22 (24.7)	12 (13.5)
Stage 3	31 (34.8)	25 (28.1)
Stage 3O	19 (21.3)	16 (18.0)
Stage 4	8 (9.0)	7 (7.9)
Stage 4O	7 (7.9)	5 (5.6)
**Management**		
MDT Involved	67 (75.3)	
LOS in days, mean (± SD)	11.85 (12.75)	
Revascularisation	6 (6.7)	6 (6.7)
Debridement	8 (9.0)	4 (4.5)
Minor Amputation	6 (6.7)	5 (5.6)
Major Amputation	9 (10.1)	8 (9.0)
ICU Admission	3 (3.4)	2 (2.2)

Abbreviations: HRFS, High Risk Foot Service; BMI, body mass index; SBP, systolic blood pressure; WCC, white cell count; CRP, C−reactive protein; MDT, multidisciplinary team; LOS, length of stay; ICU, Intensive Care Unit

Definitions: The PEDIS (Perfusion, Extent, Depth, Infection, Sensation) classification developed by the International Working Group of the Diabetic Foot (IWGDF) is a universally accepted classification system developed primarily for DFI research [11]. The PEDIS classification ranges from 1 to 4, ascending with severity, with the suffix O denoting osteomyelitis. A PEDIS score of 1 represents a foot ulcer of no infection, whilst a PEDIS score of 4 represents a severe infection with signs of a systemic response

### Primary outcomes

The primary outcome was length of stay. Length of stay for all hospitals involved was summed up to derive each participant’s total length of stay. The main secondary outcome was surgical intervention rates. Surgical outcomes were further classified into no surgery, debridement, minor amputation, or major amputation which were performed at a different tertiary hospital with a full-time vascular service.

### Primary intervention

The primary intervention for this study was MDT involvement. To identify patients who had MDT involvement during their admission, the scope of MDT had to be properly defined. In Australia, the standard inpatient MDT for DFI is made up of endocrinology, vascular surgery, podiatry, wound care nursing, and infectious diseases [6, 12]. Generally, patients admitted with DFI at Campbelltown Hospital are admitted under endocrinology but can be admitted under general medicine if there are multiple active medical issues. Notably, the HRFS MDT at Campbelltown Hospital, operates as a consult service that formally convenes once weekly to collectively review patients, discuss opinions, and reach a consensus on treatment plans. In this study, MDT involvement was defined as the active participation of two or more of the aforementioned specialties involved in the patient’s treatment.

#### Ethical approval and consent to participate:

The study was the study is approved by the “South Western Sydney Local Health District Human Research” ethics committee (#QA18/021). The study was conducted in accordance with the Declaration of Helsinki. Informed consent was waived by the same ethics committee that approved the study (South Western Sydney Local Health District Human Research ethics committee).

### Statistical analysis

All statistical analysis was conducted using IBM SPSS Statistics Subscription Version 27 (IBM Inc. Chicago, IL, USA). Chi-square test was used for categorical variables and one-way analysis of variance (ANOVA) was used for continuous variables. The effect of surgical intervention, and MDT involvement on LOS was determined using ANOVA. The impact of year of admission on the relationship between these outcomes was analysed. Statistical significance was defined as p < 0.05. Results were presented descriptively as mean (standard deviation), ranges (IQR), counts and/or proportions where necessary.

## Results

Table 1 presents the sociodemographic characteristics of the sample population. A total of 89 hospital admissions (78 patients) were identified from the ICD-10 search over the three-year study period. Of the 89 admissions, 67 admissions had MDT involvement (75.3%); 3.4% of the sample population was indigenous and 69.7% of the sample population was known to the HRFS prior to their admission. Further patient characteristics including age, gender, medical comorbidities, ulcer characteristics, laboratory results and surgical intervention are detailed in Table 1. Comparison of MDT involvement to these variables are presented in Table 2.

**Table 2:** Comparison of demographic variables and risk factors by multidisciplinary team (MDT) involvement

Variables	MDT Involvement		
Demographics	Yes (n = 67)	No (n = 22)	*P*-value
	n,%	n,%	
Sex			
Male	49 (73.1)	15 (68.2)	0.785
Female	18 (26.9)	7 (31.9)	0.785
Indigenous (yes)	2 (3.0)	1 (4.5)	0.725
Known to HRFS (yes)	47 (70.1)	15 (68.2)	0.862
**Comorbidities**			
Obesity (BMI > 30)	45 (68.2)	16 (76.2)	0.485
Hypertension (SBP > 130mmhg)	42 (62.7)	14 (63.6)	0.936
Established Cardiovascular Disease	49 (73.1)	14 (63.6)	0.426
Chronic Kidney Disease	39 (58.2)	7 (31.8)	0.048*
End stage renal disease	9 (13.4)	1 (4.5)	0.252
Existing Foot Deformity	28 (41.8)	9 (40.9)	0.942
Peripheral Neuropathy	44 (65.7)	15 (68.2)	0.829
HbA1c % mean (SD)	8.4 (2.0)	8.2 (2.7)	0.701
HbA1c > 7% (53mmol/mol)	44 (67.7)	13 (68.4)	0.952
Current Smoker	15 (22.4)	3 (13.6)	0.543
Current Drinker	12 (17.9)	3 (13.6)	0.754
**Lab**			
WCC (> 10 × 10^9/L)	43 (64.2)	10 (47.6)	0.207
CRP (> 4.9 mg/L)	64 (95.5)	19 (95.0)	0.922
Blood Culture Performed	38 (56.7)	6 (27.3)	0.026*
Wound Culture Performed	48 (71.6)	14 (63.6)	0.594
Imaging Performed	63 (94.0)	17 (21.3)	0.038*
**PEDIS Classification**			0.172
Stage 1	2 (3.0)	0 (0.0)	
Stage 2	12 (17.9)	10 (45.5)	
Stage 3	25 (37.3)	6 (27.3)	
Stage 3O	16 (23.9)	3 (13.6)	
Stage 4	7 (10.4)	1 (4.5)	
Stage 4O	5 (7.5)	2 (9.1)	
**Management**			
Revascularisation Performed	6 (9.0)	0 (0.0)	0.146
Debridement Performed	4 (6.0)	4 (18.2)	)
Minor Amputation Performed	5 (7.5)	1 (4.5)	)0.277
Major Amputation Performed	8 (11.9)	1 (4.5)	)

*P* values are comparisons between those who did and did not have multidisciplinary team involvement during admissions

Abbreviations: HRFS, High Risk Foot Service; BMI, body mass index; SBP, systolic blood pressure; WCC, white cell count; CRP, C−reactive protein

Definitions: The PEDIS (Perfusion, Extent, Depth, Infection, Sensation) classification developed by the International Working Group of the Diabetic Foot (IWGDF) is a universally accepted classification system developed primarily for DFI research [11]. The PEDIS classification ranges from 1 to 4, ascending with severity, with the suffix O denoting osteomyelitis. A PEDIS score of 1 represents a foot ulcer of no infection, whilst a PEDIS score of 4 represents a severe infection with signs of a systemic response

From 2019 to 2021, the number of admitted patients for DFI as a primary diagnosis were (n = 24, 27% in 2019 vs. n = 28,31.5% in 2020 vs. n = 37, 41.6% in 2021; *P* = 0.181). The proportion of admissions which had MDT involvement was similar (p = 0.173) across the three years from 62.5% to 2019 (n = 15), to 75% in 2020 (n = 21), and 83.8% in 2021 (n = 31).

### Risk factors

Table 1 shows that our sample population had a high level of comorbidities for DFI including pre-existing cardiovascular disease (peripheral vascular disease, ischemic heart disease and/or cerebrovascular disease, existing foot deformity (Charcot foot, previous amputations, clawed/hammer toes, rheumatoid arthritis and/or bunions, peripheral neuropathy, chronic kidney disease, and obesity. All admissions had type 2 diabetes, 64% an HbA1c > 53 mmol/mol (n = 57, 64%), 75.3% were prescribed oral anti-diabetes medication (including 16.9% SGLT2 Inhibitors) and 48.3% were prescribed insulin.

MDT involvement was similarly distributed in both sexes (76.6% of males, and 72% of females (p = 0.785) and had no significant impact on mean HbA1c levels (%) (8.4 ± 2.1, *P* = 0.701). Patients with chronic kidney disease were more likely to receive MDT involvement during their admission than any other risk group (84.8%, p = 0.048).

### Investigations performed

Table 1 shows that of the 89 admissions, 89.9% received imaging, 49.4% a blood culture, and 69.7% a wound culture. Overall, 59.6% had a raised white cell count and 93.3% an elevated CRP.

When the MDT was involved, it was significantly more common for patients to receive imaging (78.8% v 21.3%, p < 0.05). However, MDT involvement was significantly less common with patients who had a blood culture during their admission (p = 0.026).

### Length of stay

**Fig. 1:**
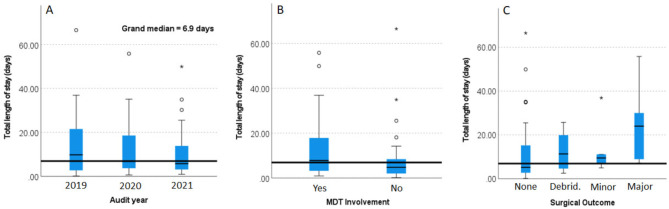
Median length of stay for participants with diabetes foot infection by (**a**) their year of admission, (**b**) multidisciplinary team (MDT) involvement (1 indicates MDT involvement), and (**c**) surgical outcomes. For surgical intervention: 0 indicates no intervention, 1 = debridement, 2 = minor amputation and 3 = major amputation

Figure 1 presents box plots of the median LOS and their interquartile ranges (IQR) by (a) the year of audit, (b) MDT involvement and, (c) surgical outcomes of participants in this study. Although the median length of stay in 2019 (9.8 days IQR = 20.2 days), 2020 (6.8, IQR 15.3 days), and 2021 were similar (5.7, IQR 10.9 days, *P* = 0.514). there was a trend towards a reduction in median LOS at an average rate of approximately 2.0 days a year.

With MDT involvement, the average length of stay was 12.4 days (SD = 11.9) and without MDT involvement the average length of stay was 10.1 days (SD = 15.3).

For those who required major amputation, median LOS was 24 days (IQR 21.5) which was significantly higher than those managed conservatively (no surgical intervention), 5.2 days (IQR 13.0) (difference 18.8 days, *P* < 0.05).

Days of delay (in inter-facility transfer) were not statistically significant between the three years. There were 16 patients who experienced delays to surgery due to hospital bed limitations with an average duration of delay to surgery being approximately 1.4 days.

### Surgical outcomes

A total of 66 admissions (74.2%) were managed conservatively and 23 admissions (25.8%) required surgical intervention. Of the admissions requiring surgery, major amputation was the most common surgery (removal of the foot or part of the leg) (n = 9, 39.1%), followed by surgical wound debridement (removal of tissue) (n = 8, 34.7%) and minor amputation (removal of a digit/s) (n = 6, 26%). Revascularisation procedures (angioplasty or bypass) that were performed during the hospital stay occurred in only 7% of admissions.

Overall rates of surgical intervention were similar between patients who received MDT care versus those who did not (non-MDT) (74.6% vs. 72.7%, p = 0.262). The main difference between the non-MDT and MDT groups was in the rate of major amputations (n = 8/67, 11.9% vs. n = 1/22, 4.5%, respectively, *P* = 0.32).

## Discussion

Whilst the current literature on multidisciplinary teams and their role in the management of DFI suggests that MDTs improve the health outcomes of patients [7–10, 13], the impact of a MDT on LOS and the degree of surgical intervention is not well established. This audit aimed to determine the effect of MDT involvement had on LOS and surgical outcomes of DFI patients.

The MDT model is globally recognised as the standard of care for the prevention and management of DFI [6, 12, 14] and has been adopted as the standard of care within Australian evidence based guidelines for diabetes-related foot disease since 2021 [15]. However, within Australia, the literature regarding the impacts of MDT involvement on LOS for DFI is not adequately researched and there are a lack of specific targets regarding LOS or rates of amputations within current Australian guidelines [15]. Most previous studies on this topic primarily originate from other countries. In Spain, a retrospective cohort study reported a statistically significant reduction in LOS and lower extremity amputation following the implementation of an MDT [7]. Similarly, in Korea, a cross-sectional study reported a reduction of almost 50% in the mean LOS for DFI patients who received MDT care when compared to a non-MDT cohort [16].

In our study, we found that the median LOS for patients with DFIs was similar throughout the three years of the study duration with no significant reduction. However, the median LOS trended downwards at an average rate of approximately 2.0 days per year but did not reach statistical significance. Benchmark figures for comparing the LOS for DFI admissions are limited in an Australian inpatient setting. One Australian study of two tertiary hospitals in Sydney from 2012 to 2017 provides some context. It reports a median LOS of 8–10 days for inpatient admissions related to diabetic foot ulcers [17] which aligns with our findings of a median LOS of 6–10 days, irrespective of MDT involvement. Interestingly, this study found that being known to a local HRFS MDT did not predict a reduction in cumulative LOS. However, it found that LOS was significantly longer for males, older people and those with increasing comorbidities, but was significantly shorter in patients with podiatry attendance [17].

When comparing our findings with other similar overseas studies that used mean LOS, our findings differed with a longer LOS in patients who had MDT involvement than those who did not (12.4 ± 11.9 days versus 10.1 ± 15.3 days) [7, 16]. A possible explanation for these differences was perhaps that patients who required MDT input had far more complex medical comorbidities, and therefore required additional involvement from other medical teams. For example, patients with chronic kidney disease were significantly more likely to need MDT care, which increases the complexity of the admission and subsequently, the LOS, thus potentially biasing results.

Furthermore, it is well established that the prevalence of DFI is considerably higher in individuals with low socioeconomic status as these individuals face more barriers in accessing various health services, including those necessary for DFI management and preventative care [14]. Additionally, the presence of low health literacy amongst individuals with diabetes is associated with poor management of their disease with higher self-reported complications of diabetes [18]. The demographic of our study population was from an area of both high socioeconomic disadvantage and low health literacy [2]. A combination of low socioeconomic status (SES) and poor health literacy can lead to delays in seeking care, non-compliance with treatment, poor self-management of diabetes, improper foot care and consequently lead to more severe infections that require prolonged hospital stays. Additionally, it may be worth noting that low SES and poor health literacy, which often result in ineffective self-care and disease management at home, could be a useful indicator of potential hospital re-admission in future studies, and highlights the importance of diabetes education to all patients with DFIs to prevent this. It is likely that the disadvantaged backgrounds of this population are potential factors that contribute to the infection severity, burden of disease and consequently, complexities of the patients who present for DFI.

Additionally, as this was single centre study, the sample size of our study was considerably smaller than other international studies. Therefore, it is worth noting that whilst we found no statistically significant relationship between MDT involvement and reducing the LOS, it is likely that other factors such as individual patient characteristics may have played a role in the LOS results.

Over the study period, we observed a 54.2% increase in the number of admissions. Although this was not statistically significant, it could be related to several factors such as the increase in the prevalence of disease in the population, increased awareness of DFI within the population and better identification of high-risk patients within the ED or clinic. This underscores the significance of addressing and managing DFI effectively to prevent hospital admissions. Despite the increased number of admissions, the proportion of HRFS MDT involvement also rose throughout these three years, at a rate of approximately 11% per year and demonstrates the shift in management paradigms towards MDT, particularly the newly augmented HRFS, as the standard for DFI management.

When looking at the effects of MDT involvement on surgical outcomes, past studies suggest that MDTs reduce the number of lower extremity amputations and revascularisation procedures required. A 2015 study conducted in an Australian tertiary hospital observed a significantly reduced number of lower extremity amputations in patients who had MDT involvement [10]. Similarly, a more recent systematic review of 33 studies found that 94% of studies reported a reduction in major amputations after instituting a diabetic foot multidisciplinary team [13].

In our study, the proportion of patients who did and did not receive surgery in both MDT and non-MDT groups was similar. However, when looking at those who had surgical interventions in both groups (n = 15), patients who had MDT care had a higher rate of major amputation (8/15, 53.3%) compared to the non-MDT group (1/15, 6.7%), suggesting that inpatient MDT involvement plays a role in early intervention through major amputations. Whilst this finding aligns with current research [9, 10, 13], the difference did not reach significantly significance, likely due to insufficient statistical power of our small sample size.

However, whilst the findings mentioned above are not statistically significant, they are important in providing context for one of our key findings, which showed that patients who required major amputations had a significantly longer LOS than those managed conservatively.

Revascularisation procedures are performed to improve wound healing and reduce the risk of amputations in patients with DFI [19]. In our study, 7% of admissions received a revascularisation procedure during their admission. This rate is notably low when compared to other tertiary hospitals in the area, as seen in similar tertiary hospitals in Sydney reporting a revascularisation requirement rate of 27% in their diabetic foot patients between 2014–2018 [20]. The limited access to vascular surgery services, available only as a half a day per week consultant review through the HRFS MDT, is a potential explanation for this disparity as it limits the number of vascular investigations or procedures that can be performed during a patient’s admission. All patients requiring revascularisation and amputation procedures had to be transferred to nearby tertiary hospital for their procedure. Additionally, patients from this population are generally of lower socio-economic status and often have trouble accessing private vascular surgery input.

Furthermore, our overall rates of patients who required minor and major amputations (n = 6, 6.7% and n = 9, 10.1% respectively) were also relatively lower when compared to the benchmark figures from previously mentioned study [20]. Yet interestingly, the rates of major amputations were higher than rates of minor amputations in our population. This suggests the presence of more severe infections requiring extensive surgical interventions, potentially due to a combination of factors such as limited vascular surgery services, delays in accessing care, low SES, health literacy issues and more.

Given that our findings show a lower-than-average revascularisation rate but one in four patients still required surgical intervention (debridement, minor amputation, or major amputation), it raises an important consideration for the need of a dedicated vascular service at Campbelltown hospital and improvements in our healthcare infrastructure. This could potentially increase the rate of revascularisation on-site, reduce the incidence of severe amputations, and ultimately improve patient outcomes.

Adhering to established guidelines is essential for optimising patient outcomes, particularly in patients with DFI [1]. Current Australian guidelines on DFI care suggest investigations such as CRP, probe to bone and plain x-rays are ordered as part of routine assessment pathway, whilst blood cultures are not ordered unless clinically indicated (Grade 4 Infection) [6]. Most patients received the recommended investigations, including imaging (n = 80, 89.9%), CRP (n = 87, 97.8%) and WCC (n = 88, 98.9%). When the HRFS MDT was involved, patients were significantly more likely to receive imaging and significantly less likely to have a blood culture during their admission. These findings indicate that the MDT approach to DFI management aligns with current guidelines, emphasising the importance of adhering to evidence-based management to ensure comprehensive care and improved patient outcomes.

### Limitations and strengths

This study had several limitations. The retrospective study design meant that the data were largely reliant on data recording. The data were not easily accessible, medical records were often inconsistent and there was a significant amount of incomplete data such as diabetes history or HbA1c. Furthermore, as with retrospective studies, reliance on historical data can increase the risk of unknown bias which affects reliability and reproducibility of the study. Another limitation was the small sample size of the audit because it was conducted at a single hospital, limiting the number of eligible participants, and reducing the statistical power. The few surgical interventions also made it less meaningful to calculate the high-low amputation ratio as suggested by Wrobel et al [21]. The study duration was also relatively short compared to international studies [7–9, 16] and the long term impacts of augmenting the HRFS MDT may not have been fully captured. The unavailability of the creatinine data meant we were unable to report on the impact of MDT on this variable. Future studies involving a population sample of a state-wide or national level over a longer period and including some other variables such as creatinine would improve the statistical power and validity of results.

A major confounding factor in assessing LOS as a main outcome in patients with DFI is that diabetes is a systemic disease, and therefore other end-organ complications (e.g., renal or cardiac issues) can skew the LOS of patients initially admitted for DFI. To adjust for this in a multivariable analysis, a larger sample size with more associated data is necessary. But within the limited scope of this audit, it was not possible to easily quantify whether non-DFI factors may have skewed LOS outcomes.

For future studies regarding this topic, a large-scale randomized control trial of MDT input would be the most effective study design, however we recognise the ethical issues that may arise from withholding the MDT care model for DFI patients. A similar retrospective study with a larger sample size, longer study duration and a focus on specific patient characteristics may help provide a more robust and comprehensive understanding of the relationship between MDT involvement, LOS and, surgical interventions in DFI patients in Southwestern Sydney.

## Conclusion

This study investigated the impact of an augmented MDT on LOS and surgical outcomes of patients with DFI at a tertiary Sydney hospital. The median LOS and rate of surgical outcomes remained similar in DFI patients who received MDT care and those that did not. However, our below-average revascularisation rate yet significant surgical intervention requirements, highlights the necessity for a dedicated vascular service at Campbelltown Hospital. HRFS-MDT involvement was more prevalent in patients with serious comorbidities such as CKD and therefore, was associated with more thorough investigation of DFI. Given the study’s shorter duration compared to most research in this area, the long-term impact of the HRFS-MDT involvement has yet to be fully captured. Overall, this study represents a useful report of DFI outcomes in a priority population area, with different healthcare resources and needs to those previously reported in Australia. For clinicians, the study identifies key areas for clinical practice improvement (including the value add of MDT care) and from a health policy standpoint, demonstrated a clear need to consider equity in access to care. However, further research is needed to appreciate the impact of MDT care on LOS and surgical outcomes in DFI patients in SWS, to provide insights for the development and optimisation of the MDT approach in managing DFI.

## Data Availability

The dataset supporting the conclusions of this article is included within the article.

## Abbreviations

ANOVA: Analysis of Variance
BMI: Body Mass Index
BP: Blood Pressure
CRP: C-reactive Protein
CVD: Cardiovascular Disease
DFI: Diabetic Foot Infection
ED: Emergency Department
eMR: Electronic Medical Records
ESR: Erythrocyte sedimentation rate
HR: Heart Rate
HRFS: High Risk Foot Service
ICD-10: International classification of diseases, tenth edition
IWGDF: International Working Group on the Diabetic Foot
LOS: Length of Stay
MDT: Multidisciplinary Team
PEDIS: Perfusion, Extent, Depth, Infection, Sensation
RR: Respiratory Rate
SD: Standard Deviation
SWSLHD: Southwestern Sydney Local Health District
WCC: White Cell Count
